# Cell-Free DNA Variables including Gene Mutations in CA15-3 Normal Breast Cancer Reflect Prognosis

**DOI:** 10.1155/2022/5470166

**Published:** 2022-02-24

**Authors:** Juan Xu, Wenxiu Chen, Ziwei Sun, Hailin Peng, Chenglin Zhou, Shiyang Pan

**Affiliations:** ^1^Department of Laboratory Medicine, The First Affiliated Hospital of Nanjing Medical University, Nanjing 210029, China; ^2^Department of Laboratory Medicine, Taizhou People's Hospital, Taizhou 225300, China

## Abstract

**Background:**

Cell-free DNA (cfDNA) has attracted considerable attention in precision medicine. However, few data are available regarding to the prognostic value of cfDNA variables in CA15-3 normal breast cancer (BC) patients. Here, we aimed at investigating the prognostic value of cfDNA variables including gene mutations in CA15-3 normal BC patients.

**Methods:**

A total of 68 BC patients with normal CA15-3 levels were enrolled. cfDNA concentration and integrity were assessed based on qPCR. cfDNA gene mutations were conducted by using next gene sequencing (NGS). The association between cfDNA variables and the prognosis of patients was analyzed.

**Results:**

cfDNA concentration was related to tumor stage (*P* = 0.002), metastases (*P* = 0.001), and distant metastases (*P* < 0.001). The elevated copy number variants (CNV) were found in distant metastasis patients compared with patients without distant metastases (*P* = 0.008). Nineteen mutant genes were validated in enrolled CA15-3 normal BC patients. Thirty-two patients (47.0%) had single nucleotide variants (SNV), and 13 (19.1%) patients had *TP53* mutations (*TP53*^mut^). SNV (*P* = 0.033) was related to tumor stage, and *TP53*^mut^ was related to metastases (*P* = 0.016) and distant metastases (*P* = 0.006). In multivariate logistic analysis, cfDNA concentration was associated with metastases (OR = 3.404, 95% CI: 1.074-10.788, *P* = 0.037) and distant metastases (OR = 13.750, 95% CI: 1.473-128.358, *P* = 0.021). Cases with high cfDNA levels (>15.6 ng/ml), SNV, and *TP53*^mut^ showed worse DFS compared with patients with low cfDNA levels (*P* < 0.001), without SNV (*P* = 0.002) and with *TP53* wildtype (*P* < 0.001), respectively. In the multivariate Cox proportional hazard model, cfDNA concentration was an independent predictor of poor survival (HR = 5.786, 95% CI: 1.101-30.407, *P* = 0.038).

**Conclusions:**

Assessment of cfDNA concentration, CNV, SNV, and *TP53*^mut^ could be useful in predicting prognosis for CA15-3 normal BC patients. The cfDNA concentration was an independent predictor prognostic factor in CA15-3 normal BC patients.

## 1. Introduction

Breast cancer (BC) is the most frequently diagnosed cancer all around the world. In 2020, more than half a million people died from this disease worldwide [1]. In China, BC ranks first in terms of incidence and fifth in terms of mortality among all cancers in women, and the burden of BC incidence and mortality is rapidly growing [2]. BC patient prognosis is mainly determined by several clinical characteristics, such as stage, metastasis, and molecular subtypes [3]. To improve survival prediction for BC patients, researchers are ongoing on the search for new prognostic markers. Cancer antigen 15-3 (CA15-3) is overexpressed in multiple cancers and is known as a representative biomarker for malignant tumor [4]. In clinical routine, utilizing CA15-3 for predicting BC prognosis has been widely incorporated [5]. However, in a study of 1046 BC cases by Ebeling et al., CA15-3 was not identified as an independent prognostic biomarker [6]. Another study also reported no association between CA15-3 levels and survival outcome in BC patients younger than 40 years old [7]. Moreover, blood biomarkers were often measured for evaluating prognosis of BC patients, but they were few measured in populations with normal CA15-3 levels. Therefore, new noninvasive prognostic markers for BC patients with normal CA15-3 levels to assist predicting the survival outcome are urgently needed.

Recently, liquid biopsy has been developed fast because of the advantage of noninvasiveness and the potential to reflect clinically relevant information of tumor [8]. It has been shown that liquid biopsy, such as cell-free DNA (cfDNA), microRNA, and circulating tumor cells, can be evaluated as potential prognostic biomarkers in BC [9–12]. Among these biomarkers, cfDNA has attracted considerable attention. cfDNA concentrations, which represent the quantity of cfDNA, are elevated in various cancers compared with levels in healthy controls [13, 14]. cfDNA integrity (cfDI), which indicates the quality of cfDNA, is based on the ratio of longer DNA fragment to shorter ones of a specifc genetic elements, such as long interspersed nuclear element 1 (LINE1). cfDNA variables including concentration and integrity have been proposed as prognostic oncological biomarkers [15, 16]. However, usefulness of these cfDNA variables in CA15-3 normal BC patients has so far not been shown.

Approximately 10% of BC cases are related to genetic mutations. Mutations in the *BRCA1* and *BRCA2* genes are the most common high-risk mutations associated with BC [17]. Gene mutations in cfDNA have shown some prognostic significance, and these mutations can be detected in plasma cfDNA by next generation sequencing (NGS) [1]. There is a clear correlation between presence of *TP53* mutations in cfDNA and adverse progression-free survival (PFS) outcome [18]. Several retrospective studies have also reported that *ESR1* or *PIK3CA* mutations were related to shorter PFS and overall survival (OS) in hormone receptor- (HR-) positive metastatic BC [19].

In this study, we analyzed cfDNA variables including gene mutations in CA15-3 normal breast cancer. Gene mutations in cfDNA were confirmed by using NGS on a 50 genes panel. Our results showed that cfDNA concentration, copy number variation (CNV), single nucleotide variant (SNV), and *TP53* mutations had the potential to predict the survival of CA15-3 normal BC patients. High concentration of cfDNA was an independent predictor of poor survival in CA15-3 normal BC patients.

## 2. Patients and Methods

### 2.1. Patients

The study included 68 women identified from Taizhou People's Hospital and diagnosed with BC with normal CA15-3 levels between August 2018 and August 2019. The diagnoses of all patients were confirmed by core biopsy. Blood samples were collected before surgery, and CA15-3 levels were detected by electrochemiluminescence. CA15-3 levels were determined by standard values for our institution: normal CA15-3 level was defined as serum CA15-3 below the cut-off value of 31.3 U/ml; elevated CA15-3 level was defined as serum CA15-3 above the cut-off value of 31.3 U/ml. Patients with elevated CA15-3 levels were excluded. Follow-up information and clinical information were obtained using the institutional database. The last update of clinical information was completed on August 31, 2020. This study was conducted in accordance with the Declaration of Helsinki on 2013. Informed consent was obtained from all patients, and this study was approved by the ethics committee of the Taizhou People's Hospital (KY 201804801, Taizhou, China).

### 2.2. Sample Processing

Plasma samples were obtained from BC patients at the time of diagnosis, prior to surgery or therapy. Blood samples (2-3 ml) were collected in EDTA tubes and processed immediately. Plasma components were separated by centrifugation at 1600 g for 10 minutes at 4°C. Plasma was then transferred to new 1.5 ml EP tubes and centrifuged a second time at 12000 g for 10 minutes at 4°C. The plasma was stored at -80°C until the time of DNA extraction.

### 2.3. Cell-Free DNA Extraction

Plasma was thawed on ice first, and then it was centrifuged at 12000 g for 5 minutes before DNA extraction. cfDNA was extracted from about 1 ml plasma using the CWhipro Circulating DNA Midi Kit (CWBIO, Beijing, China) in accordance with the manufacturer's instructions. Extracted cfDNA was eluted in 40 *μ*L elution buffer, and the final eluted cfDNA was immediately used for analysis or stored at -20°C.

### 2.4. Concentration and Integrity of cfDNA Measurement

The short and long DNA fragments of LINE1 (LINE1-97 bp and LINE1-259 bp) were measured by qPCR as previous study [20]. In brief, concentration of cfDNA was represented with LINE1-97 base pair (bp) fragment qPCR result (LightCycler LC480, USA). cfDI was based on the ratio of long and short fragment of the LINE1 element: LINE1-259/97. A reference standard curve was created by a serially diluted standardized solution of human genomic DNA (Thermo Fisher, USA). The LINE1-97 bp qPCR fragment was amplified using the forward (5′-AGGTGCTGGAGAGGATGT-3′) and reverse (5′-GGAATCGCCACACTGACT-3′) primer; the LINE1-259 bp fragment was amplified using the forward (5′-TGCCGCAATAAACATACGTG-3′) and reverse (5′-AACAACAGGTGCTGGAGAGG-3′) primer.

### 2.5. Analysis of cfDNA by NGS

By using the Oncomine cfDNA assay system (Thermo Fisher Scientific, USA), we constructed the library for cfDNA sequencing. This assay system can detect 50 genes (see Table 1) with 207 amplicons including 2855 hotspots by using 10 ng of cfDNA.

cfDNA was used to generate libraries using Fast cfDNA Library Prep Set for MGI (CWBIO, China) following the manufacturer's instructions. Library circularization and MGI sequencing (MGISEQ-2000) were performed using the Circularization Kit for MGI (CWBIO, China) and MGISEQ-2000RS library Sequencing Kit (PE100, BGI), independently. Variant caller was configured to call high stringency somatic variants, and the hotspot_min_allele_frequency was set to 0.01. ANNOVAR was used to annotate all variants. All data were manually reviewed to provide a precise interpretation by using the Integrated Genomics Viewer package (v2.3.25).

### 2.6. CNV Detection

Burrows-Wheeler Aligner software was used to perform alignment analysis for raw reads. Sequences that could be mapped to just one location in the hg19 reference human genome without mismatch were counted. GC bias correction was performed as described elsewhere before [21]. The read counts for 500 kb bin size and in each 0.5% of GC content bin were both got. ^−^*M* and *M*_*i*_ were the average counts across all GC content bins and in bin *i*, respectively. Counts in each GC content bin were weighted by *W*_*i*_ = ^−^*M*/*M*_*i*_. Counts ratio was normalized by 500 kb bin counts/all counts, and a reference baseline was created. *Z* score was calculated by the following: (sample counts ratios − mean of reference counts ratios)/standard deviation of reference counts ratios. For data visualization, we further plotted *Z* score into genome-wide copy number by using ggplot2 [21]. The marker of CNVZ was scored by the formula avg. (abs (*Z* score)).

### 2.7. Statistics

Statistical analysis was performed using SPSS 22.0 software (IBM, USA). The independent-samples *t-*test, Mann–Whitney *U* tests, and one-way ANOVA analysis were used, where appropriate, to evaluate the significance of differences among different groups. Association between variables and clinical characteristics was evaluated by chi-square or Fisher exact test. Univariate and multivariate logistic regression analysis were performed to identify odds ratios (OR) for metastases and distant metastases. Disease-free survival (DFS) referred to the period between the date of surgery and the date of progression, last follow-up, or death. Kaplan-Meier analysis was used to plot survival curves, and the differences between survival curves were determined by log-rank test. A Cox regression model was utilized to evaluate hazard ratios (HR) for DFS. *P* < 0.05 was considered statistically significant.

## 3. Results

### 3.1. Patient Characteristics

Sixty-eight BC patients with normal CA15-3were enrolled in this study. The median age of patients was 50 years which ranging from 29 to 85 years (see Table 2). The majority of tumor cases had negative HER2 status (70.6%), and 14 cases (20.59%) had positive HER2 status with ER or/and PR positive. Six cases (10.9%) had been diagnosed as triple-negative BC. Among the overall patients, 31 (45.6%) BC patients had a tumor size ≤ 2 cm while 37 (54.41%) cases had a tumor > 2 cm. Furthermore, 41 cases (60.3%) were diagnosed with stage I/II BC, and 27 cases (39.7%) were diagnosed with stage III/IV BC. Thirty-six patients (52.9%) had metastases, and 13 patients (19.1%) had distant metastases. The median follow-up time was 12 months (range 3-22 months).

### 3.2. Association between cfDNA Concentration, Integrity, and CNV with Clinical Characteristics

The distributions of clinical characteristics factors according to concentration, integrity, and CNV of cfDNA are shown by in Table 3. The median cfDNA concentration, integrity, and CNV were 15.6 ng/ml (3.78-152.66), 0.23 ng/ml (0.01-1.61), and 1.09 ng/ml (0.09-2.49), respectively. The difference in the cfDNA concentration was not found according to age, molecular subtypes, and tumor size. However, cfDNA concentration tended to be higher in patients with stage III/IV (25.02 (12.67, 35.30)) compared with patients with stage I/II (13.76 (8.94, 18.85)) (*P* = 0.002). Patients with metastatic (26.16 (15.25, 42.83)) and distant metastatic status (33.62 (22.95, 41.09)) exhibited a higher concentration of cfDNA compared with patients without metastases (12.72 (9.06, 18.18)) or distant metastases (12.94 (9.41, 19.53)) (*P* = 0.001, *P* < 0.001). There was no difference in the cfDI according to clinical characteristics. The distant metastatic group (1.40 ± 0.12) had a higher CNV index compared with the group without distant metastases (1.02 ± 0.06) (*P* = 0.008).

### 3.3. Association between cfDNA Gene Mutations and Clinical Characteristics

**Figure 1:**
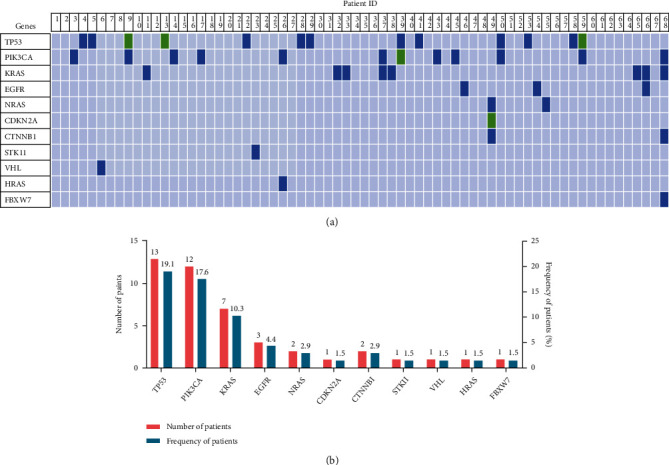
Exiting cfDNA mutations in BC patients with normal CA15-3 levels. (a) Mutation spectrum of each patient. Blue depicts the number of mutations < 2, and green depicts the number of mutations ≥ 2. (b) The histograms represent the number and frequency of BC patients with gene mutations.

Gene mutations in cfDNA were observed in 32 (47.0%) of the 68 enrolled CA15-3 normal BC patients. Mutations were identified in *TP53* (19.1%), *PIK3CA* (17.6%), *KRAS* (10.3%), *EGFR* (4.4%), *NRAS* (2.9%), *CDKN2A* (1.5%), *CTNNB1* (2.9%), *STK11* (1.5%), *VHL* (1.5%), *HRAS* (1.5%), and *FBXW7* (1.5%). Overall, 9 patients harbored more than two mutated genes (see Figure 1). Patients with advanced stage (17/27) were more likely to have a high amount of SNV compared with stage I/II patients (15/41) (*P* = 0.033). *TP53* mutations were more frequent in the metastatic (10/32) and distant metastatic (6/13) groups relative to the nonmetastatic (3/36) (*P* = 0.016) and nondistant metastatic group (7/55) (*P* = 0.006), respectively (see Table 4).

### 3.4. Association between cfDNA Variables and BC Prognosis

A strong relationship between cfDNA concentration and metastatic status was observed both in univariable analysis and multivariable logistic regression analysis (OR = 3.404, 95% CI: 1.074-10.788, *P* = 0.037) (see Table 5). Logistic regression analysis also confirmed that high cfDNA concentration was associated with distant metastatic status in enrolled CA15-3 normal BC patients (OR = 13.750, 95% CI: 1.473-128.358, *P* = 0.021) (see Table 6).

**Figure 2:**
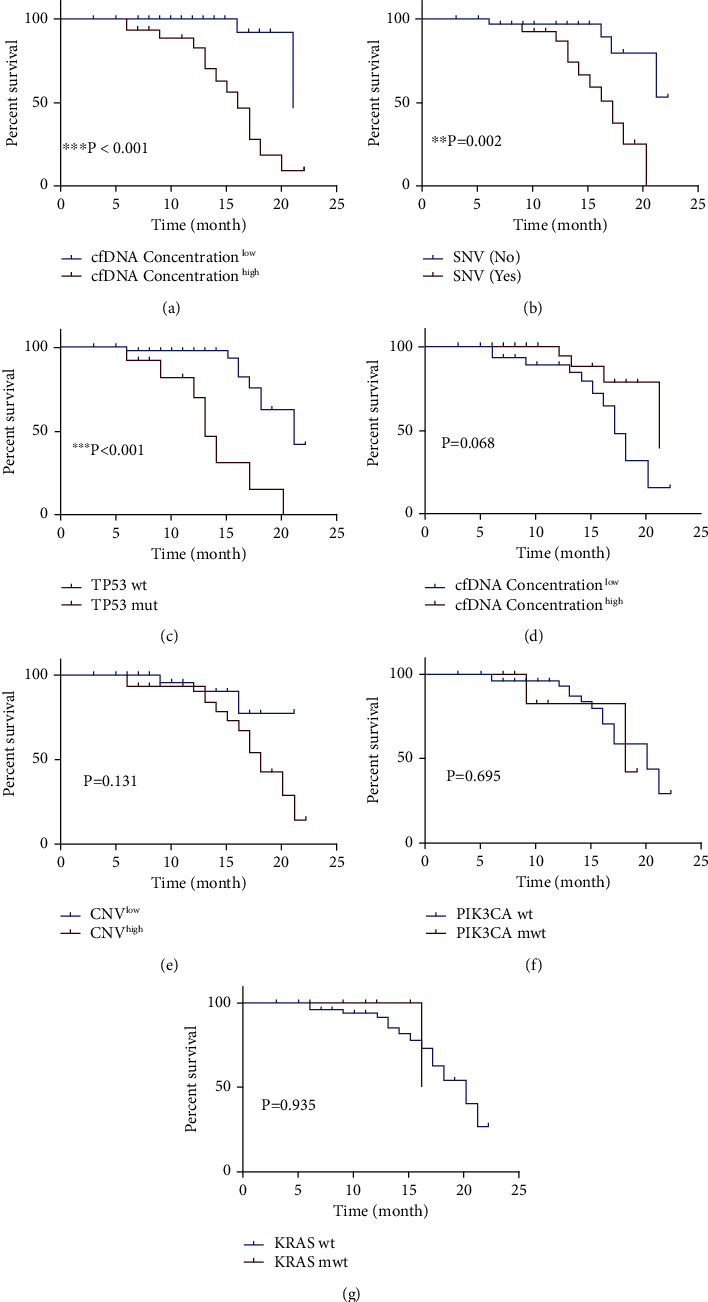
Kaplan-Meier DFS curves based on cfDNA of BC patients (*n* = 68). (a) cfDNA concentration. (b) SNV. (c) *TP53* mutations. (d) Integrity. (e) CNV. (f) *PIK3CA* mutations. (g) *KRAS* mutations.

Patients were divided into two groups (high and low) according to the concentration of cfDNA. High concentration of cfDNA (>15.6 ng/ml) was associated with a poor DFS (see Figure 2(a), *P* < 0.001). In patients with SNV, DFS tended to be shorter (see Figure 2(b), *P* = 0.002). Patients with *TP53* mutations showed a shorter DFS compared with those without *TP53* mutations (see Figure 2(c), *P* < 0.001). There was no difference in DFS according to cfDI, CNV, and mutations of *PIK3CA* and *KRAS* (see Figures 2(d)–2(g), all *P* > 0.05). In the further Cox proportional regression survival analysis, high concentration of cfDNA was an independent predictor of poor survival (HR = 5.786, 95% CI: 1.101-30.407, *P* = 0.038) (see Table 7).

## 4. Discussion

In this study, we evaluated the association between cfDNA variables including gene mutations and the prognosis of BC patients with normal CA15-3 levels. Our results revealed an elevated level of cfDNA concentration, CNV, SNV, and *TP53* mutations in stage III/IV BC patients or patients with metastases or even distant metastases. Logistic regression analysis also showed that high cfDNA concentration was positively associated with metastatic and distant metastatic status. In the multivariate analysis, we identified that the concentration of cfDNA was an independent predictor correlated with adverse survival outcome.

BC is a distinctly heterogeneous tumor with various prognosis and is divided into four major molecular (triple-negative, HER2+, luminal-A, and luminal-B) subtypes. Nowadays, the selection of treatment is mainly based on the molecular subtype in clinical practice, and the prognosis of BC patients varies with molecular subtype [3]. Tumor biomarkers are widely used for monitoring cancer prognosis, of which CA15-3 is a conventional marker for BC. However, previous studies [22] have shown that less than 20% BC patients have elevated levels of serum CA15-3. Hence, in BC patients with normal CA15-3 levels, there was a lack of an accredited blood biomarker that assists the clinicians to monitor the outcome without recourse to expensive imaging.

Liquid biopsy is becoming more and more important in precision medicine. Many traditional biomarkers identified from liquid biopsy such as microRNA, circulating DNA, and circulating tumor cells have been investigated as prognostic markers in various kinds of tumors, including BC [8]. cfDNA variables are prominent biomarkers among them. Relationship between cfDNA variables and different molecular subtypes was reported extensively. However, as far as we know, this is the first study evaluating the prognostic value of cfDNA variables in BC patients with normal CA15-3 levels.

Studies have shown that concentration of cfDNA increases with disease progression, and monitoring dynamic cfDNA concentration is clinically important to evaluate prognosis in cancer patients. For example, Cheng et al. showed that cfDNA concentration could serve as an independent prognostic marker in metastatic BC patients [23]. Shibayama et al. found increased cfDNA concentration in BC patients with the increased number of organs with metastases [24]. These studies promised the prognosis value of cfDNA concentration in BC regardless of the CA15-3 levels. In agreement with previous studies, we focused on BC patients with normal CA15-3 levels, and our results demonstrated that the concentration of cfDNA was related with stage, metastases, and distant metastases. Furthermore, cfDNA concentration was an independent predictor of DFS of BC with normal CA15-3 levels.

As to cfDNA integrity (cfDI), it remains controversial about the association of high cfDI with poor survival outcome in BC patients. Lam and his colleagues documented that high cfDI was correlated with poor RFS in newly diagnosed BC patients [25], whereas Cheng et al. and Madhavan et al. found opposite results in patients with metastatic BC [23, 26]. DNA fragments released by apoptotic cells ranged from approximately 180 to 200 bp. On the contrary, DNA fragments released by malignant cells in cancer patients vary in length size for their undergoing different pathophysiological processes [27]. Recent studies observed short fragments of cfDNA in cancer patients compared with healthy individuals [28]. Our study showed no association of cfDI with clinical characteristics in BC patients with normal CA15-3 levels. Notably, we used LINE1-based real-time PCR to determine cfDI instead of the automated gel electrophoresis used in previous studies. The differences in the methodology, enrolled patient cohorts, and timing of sample collection may partly explain the different results, but more researches and systematic reviews are required to identify the prognostic potential of cfDI in BC.

Previous studies have shown that mutations in genes including *PIK3CA*, *TP53*, *ESR1*, and *ERBB2* can be used as prognostic biomarkers in BC, and these biomarkers can assist the implementation of personalized therapy for BC patients [29]. CNV and SNV are the most common variables of gene mutations related to BC [30]. A systematic review and meta-analysis revealed the potential application of cfDNA to identify the SNV and CNV in the most common genes associated with BC. They demonstrated that detecting SNV of cfDNA had a high sensitivity, specificity, and accuracy, but for CNV, there was a need for further exploration [31]. In our work, we observed significant increased levels of CNV and SNV in BC patients with distant metastases, and two gene mutations, *TP53* and *PIK3CA* mutations, were more frequently. Approximately, 19.1% of patients carried *TP53* mutations are in accordance with the previous studies, which reported the frequency of somatic *TP53* mutations ranging from 15% to 71%. Somatic *TP53* mutation leads to disruption in the cell cycle, induced apoptosis, and affected DNA damage repair process, and the presence of *TP53* mutations in cfDNA was associated with lower PFS independently of clinical treatment [18]. Approximately 17.6% of patients carried *PIK3CA* mutations. Even though the mutation frequency of *PIK3CA* was similar between invasive and ductal carcinoma in situ BC, *PIK3CA* gene mutation was reported to be driver mutation in both BC subtypes. BC patients with the *PIK3CA* mutations showed similar prognosis to patients without the *PIK3CA* mutations [32], and these findings are partly in accordance with our results in BC patients with normal CA15-3 levels.

There were several limitations in this study. First, this was a small and single institutional cohort without regarding the ethnic differences. Moreover, few recent studies focused on cfDNA variables including gene mutations in non-Asian BC patients with normal CA15-3 levels. Several studies in English suggested that analysis of cfDNA especially in combination with other biomarkers can serve as attractive prognostic for BC patients [23, 33, 34]. Other studies demonstrated that mutations detection in cfDNA may have important implications for prognosis in BC patients [35, 36]. A large-scale and multi-institutional study is required to confirm the results. In our study, 40% (27/68) BC patients with stage III-IV were enrolled. Many studies have shown that BC patients with III/IV do not have an acceptable blood marker that allows the clinician to monitor the outcome [33]. So, dedicated studies are warranted in this population. Second, the mutations were not detected in tumor because of the lack of matching tissue samples in the majority of patients. Combining mutation analysis in cfDNA and tissue samples would be a more powerful predictive marker for BC patients with normal CA15-3 levels. Third, as cfDNA variables vary from person to person, it is valuable to evaluate the gene mutations at multiple time points before and after therapy.

## 5. Conclusions

We determined cfDNA variables including gene mutations in BC patients with normal CA15-3 levels. Nineteen mutant genes were validated in enrolled CA15-3 normal BC patients. cfDNA concentration, CNV, SNV, and *TP53* mutations are shown to be prognostic predictors that associated with clinical characteristics and poor survival outcome. The concentration of cfDNA was an independent predictor prognostic factor in CA15-3 normal BC patients. These results were helpful for promoting the application of cfDNA detecting in the longitudinal monitoring of treatment management of BC patients with normal CA15-3 levels.

## Figures and Tables

**Table 1:** The 50 genes in the Oncomine cfDNA assay.

Genes	Genes	Genes	Genes	Genes
*ABL1*	*AKT1*	*ALK*	*APC*	*ATM*
*BRAF*	*CDH1*	*CDKN2A*	*EGFR*	*ERBB2*
*ERBB4*	*EZH2*	*FBXW7*	*FGFR1*	*FGFR2*
*FGFR3*	*GNAS*	*GNAQ*	*HNF1A*	*HRAS*
*IDH*	*JAK2*	*JAK3*	*IDH2*	*KRAS*
*MET*	*MLH1*	*MPL*	*NOTCH1*	*NPM1*
*NRAS*	*PDGFRA*	*PTPN11*	*RB1*	*RET*
*SMAD4*	*SMARCB1*	*SMO*	*SRC*	*STK11*
*CSF1R*	*CTNNB1*	*FLT3*	*GNA11*	*KDR*
*KIT*	*PIK3CA*	*TP53*	*PTEN*	*VHL*

**Table 2:** Clinical characteristics of the BC patients (*n* = 68).

Characteristics	Total cases, *N*	%
Age at diagnosis (years)		
≤50	32	47.06
>50	36	52.94
Molecular subtype		
Triple-negative	6	8.82
HER2^+^ (any ER/PR)	14	20.59
HER2^−^ (ER or/and PR^+^)	48	70.59
Tumor size (cm)		
≤2	31	45.59
>2	37	54.41
Tumor stage		
I/II	41	60.29
III/IV	27	39.71
Metastatic status		
No	36	52.94
Yes	32	47.06
Distant metastatic status		
No	55	80.88
Yes	13	19.12

HER2: human epidermal growth factor receptor 2; ER/PR: estrogen/progesterone receptor.

**Table 3:** Association between concentration, integrity index, CNV index, and clinical characteristics of patients (*n* = 68) (^−^*x* ± SD, *M* (*P*_25_, *P*_75_)).

Characteristics	Concentration (ng/ml)	*P*	Integrity index	*P*	CNV index	*P*
Age at diagnosis (years)						
≤50	21.74 ± 4.66	0.593	0.28 (0.12, 0.61)	0.134	1.03 ± 0.09	0.310
>50	19.20 ± 1.66		0.21 (0.08, 0.36)		1.14 ± 0.07	
ER/PR/HER2 status at diagnosis						
TNBC	34.28 ± 3.99	0.080	0.27 ± 0.09	0.695	1.14 ± 0.10	0.533
HER2^+^ (any ER/PR)	24.66 ± 9.94		0.30 ± 0.09		1.10 ± 0.15	
HER2^−^ (ER or/and PR^+^)	17.41 ± 1.47		0.32 ± 0.46		1.08 ± 0.07	
Tumor size (cm)						
≤2	18.42 ± 1.70	0.446	0.33 ± 0.05	0.658	1.01 ± 0.07	0.226
>2	22.05 ± 4.09		0.30 ± 0.06		1.16 ± 0.09	
Tumor stage						
I/II	13.76 (8.94, 18.85)	0.002^∗∗^	0.36 ± 0.05	0.141	1.03 (0.90,1.18)	0.278
III/IV	25.02 (12.67, 35.30)		0.24 ± 0.05		1.07 (0.92,1.55)	
Metastatic status						
No	12.72 (9.06, 18.18)	0.001^∗∗^	0.37 ± 0.06	0.123	1.00 ± 0.09	0.090
Yes	26.16 (15.25, 42.83)		0.25 ± 0.04		1.20 ± 0.07	
Distant metastatic status						
No	12.94 (9.41, 19.53)	<0.001^∗∗∗^	0.33 ± 0.04	0.364	1.02 ± 0.06	0.008^∗∗^
Yes	33.62 (22.95, 41.09)		0.24 ± 0.09		1.40 ± 0.12	

^∗^ *P* < 0.05, ^∗∗^*P* < 0.01, ^∗∗∗^*P* < 0.001.

**Table 4:** Association between gene mutation status and clinical characteristics of patients (*n* = 68).

Characteristics	SNV				*TP53* mutation				*PIK3CA* mutation				*KRAS* mutation			
	No	Yes	*χ* ^2^	*P*	No	Yes	*χ* ^2^	*P*	No	Yes	*χ* ^2^	*P*	No	Yes	*χ* ^2^	*P*
Age at diagnosis (year)																
≦50	16	16	0.210	0.647	23	9	3.171	0.075	25	7	0.743	0.389	31	1	2.916	0.088
>50	20	16			32	4			31	5			29	7		
ER/PR/HER2 status at diagnosis																
TNBC	2	4	1.663	0.435	4	2	2.388	0.303	6	0	3.152	0.207	4	2	3.061	0.216
HER2^+^ (any ER/PR)	9	5			13	1			14	0			13	1		
HER2^−^ (ER or/and PR^+^)	25	23			38	10			36	12			43	5		
Tumor size (cm)																
≤2	16	15	0.040	0.841	25	6	0.002	0.964	27	4	0.882	0.348	27	4	0.071	0.79
>2	20	17			30	7			29	8			33	4		
Tumor stage																
I/II	26	15	4.546	0.033^∗^	36	5	3.200	0.074	34	7	0.023	0.878	37	4	0.401	0.526
III/IV	10	17			19	8			22	5			23	4		
Metastatic status																
No	21	15	0.893	0.345	33	3	5.754	0.016^∗^	31	5	0.743	0.389	32	4	0.031	0.859
Yes	15	17			22	10			25	7			28	4		
Distant metastatic status																
No	30	25	0.297	0.586	48	7	7.598	0.006^∗∗^	44	11	1.096	0.295	49	6	0.203	0.652
Yes	6	7			7	6			12	1			11	2		

SNV: single nucleotide variant. ^∗^*P* < 0.05, ^∗∗^*P* < 0.01.

**Table 5:** Logistic regression analysis between cfDNA variables and metastatic status in BC patients with normal CA15-3 levels.

Characteristics	Univariable analysis			Multivariate analysis		
	OR	95% CI	*P*	OR	95% CI	*P*
Age at diagnosis (year)						
≤50	1.000			1.000		
>50	0.387	0.145-1.030	0.057	0.316	0.098-1.018	0.054
Tumor size (cm)						
<2	1.000					
≥2	1.572	0.698-3.537	0.275			
Stage						
I/II	1.000			1.000		
III/IV	0.840	0.317-2.228	0.726	0.813	0.247-2.678	0.733
ER/PR/HER2 status at diagnosis						
TNBC	1.000					
HER2+ (any ER/PR)	0.500	0.068-3.675	0.497			
HER2- (ER or/and PR+)	0.389	0.065-2.331	0.301			
cfDNA concentration						
Low	1.000			1.000		
High	4.400	1.588-12.193	0.004^∗∗^	3.404	1.074-10.788	0.037^∗^
cfDNA integrity						
Low	1.000					
High	0.489	0.186-1.286	0.147			
CNV						
Low	1.000					
High	2.046	0.777-5.386	0.147			
SNV						
No	1.000			1.000		
Yes	3.818	1.396-10.443	0.009^∗∗^	2.088	0.583-7.476	0.258
*TP53* mutation						
No	1.000			1.000		
Yes	8.905	1.795-44.186	0.007^∗∗^	4.536	0.730-28.206	0.105

^∗^ *P* < 0.05, ^∗∗^*P* < 0.01. Hosmer and Lemeshow Test: *P* = 0.936.

**Table 6:** Logistic regression analysis between cfDNA variables and distant metastatic status in BC patients with normal CA15-3 levels.

Characteristics	Univariable analysis			Multivariate analysis		
	OR	95% CI	*P*	OR	95% CI	*P*
Age at diagnosis (year)						
≤50	1.000			1.000		
>50	1.543	0.448-5.311	0.492	2.627	0.467-14.782	0.273
Tumor size (cm)						
<2	1.000					
≥2	2.289	0.824-6.355	0.112			
Stage						
I/II	1.000			1.000		
III/IV	1.388	0.410-4.694	0.598	0.862	0.169-4.382	0.858
ER/PR/HER2 status at diagnosis						
TNBC	1.000					
HER2+ (any ER/PR)	0.800	0.102-6.249	0.832			
HER2- (ER or/and PR+)	0.341	0.052-2.231	0.262			
cfDNA concentration						
Low	1.000			1.000		
High	18.000	2.182-148.486	0.007^∗∗^	13.750	1.473-128.358	0.021^∗^
cfDNA integrity						
Low	1.000			1.000		
High	0.232	0.058-0.938	0.040^∗^	0.285	0.055-1.462	0.132
CNV						
Low	1.000			1.000		
High	4.306	1.066-17.389	0.040^∗^	4.192	0.718-24.494	0.112
SNV						
No	1.000			1.000		
Yes	4.638	1.147-18.751	0.031^∗^	1.363	0.201-9.233	0.751
*TP53* mutation						
No	1.000			1.000		
Yes	5.878	1.526-22.633	0.010^∗^	4.154	0.596-28.951	0.151

^∗^ *P* < 0.05, ^∗∗^*P* < 0.01. Hosmer and Lemeshow Test: *P* = 0.498.

**Table 7:** Univariate/multivariate Cox proportional regression survival analysis.

Characteristics	Univariable analysis			Multivariate analysis		
	HR	95% CI	*P*	HR	95% CI	*P*
Age at diagnosis (year)						
≤50	1.000					
>50	1.800	0.610-5.309	0.287			
Tumor size (cm)						
<2	1.000					
≥2	0.799	0.342-1.862	0.603			
Stage						
I/II	1.000			1.000		
III/IV	1.091	0.376-3.165	0.873	0.346	0.088-1.355	0.127
ER/PR/HER2 status at diagnosis						
TNBC	1.000					
HER2+ (any ER/PR)	0.367	0.071-1.900	0.232			
HER2- (ER or/and PR+)	0.348	0.090-1.340	0.125			
cfDNA concentration						
Low	1.000			1.000		
High	10.204	2.291-45.437	0.002^∗∗^	5.786	1.101-30.407	0.038^∗^
cfDNA integrity						
Low	1.000					
High	0.361	0.114-1.144	0.083			
CNV						
Low	1.000					
High	2.558	0.713-9.182	0.150			
SNV						
No	1.000			1.000		
Yes	6.042	1.666-21.904	0.006^∗∗^	2.580	0.416-16.011	0.309
*TP53* mutation						
No	1.000			1.000		
Yes	7.414	2.528-21.741	0.001^∗∗∗^	2.771	0.699-10.987	0.147

^∗^ *P* < 0.05. Omnibus test: *P* < 0.001.

## Data Availability

The datasets analyzed during this study are available from the corresponding author on reasonable request.
